# Addressing fears of children with Williams syndrome: therapist and child behavior in the context of a novel play-and humor-infused exposure therapy approach

**DOI:** 10.3389/fpsyg.2023.1098449

**Published:** 2023-08-03

**Authors:** Brianna N. Young, Ellora Mohanty, Karen Levine, Bonita P. Klein-Tasman

**Affiliations:** ^1^Child Neurodevelopment Research Lab, Department of Psychology, University of Wisconsin-Milwaukee, Milwaukee, WI, United States; ^2^Department of Psychiatry, Harvard Medical School, Boston, MA, United States

**Keywords:** Williams syndrome, children, phobias, anxiety, play therapy, exposure therapy

## Abstract

Many children with Williams syndrome struggle with fears and phobias that significantly impact their daily lives. Yet, there is sparse literature about the impact of behavioral interventions to treat anxiety and phobias among children with Williams syndrome. Using observational coding of intervention videos, the current study examines patterns of the therapist's use of play and humor and relations to child behavioral responses for four children with Williams syndrome who were identified as treatment responders to humor- and play-infused exposure therapy for fears and anxieties. Sessions were coded for therapist behaviors (exposure with or without play/humor, stimulus type used during exposure, passive or invited attention to feared stimulus, and spontaneous parent participation in exposure) as well as positive, negative, and neutral child behaviors (verbalizations and behaviors). Temporal patterns between therapist and child behaviors were analyzed using lag sequential analyses. The results showed that tolerance of feared stimuli improved for two of the four children following this play- and humor-infused exposure therapy approach, and the remaining two participants demonstrated progress beyond tolerating the feared stimulus and showed increased positive behaviors with the feared stimulus across sessions. Findings also showed patterns of therapist attunement to the child's anxiety level demonstrated through efforts to flexibly adjust the degrees of exposure. Therapist-initiated invited attention behaviors, indicative of the therapist's use of narration and priming, were associated with child tolerance and positive behaviors during exposure to the feared stimulus. Limitations of this study include a very small sample size, short duration of intervention, and a single-subject research design, which limit the generalizability of findings. Implications and future directions of this research are discussed.

## Introduction

Williams syndrome is a rare neurodevelopmental disorder caused by a hemizygous microdeletion on chromosome 7q11.23 and characterized by physical, cognitive, and behavioral markers (Mervis and John, 2010). Williams syndrome has a prevalence of 1 in every 7,500 births and with roughly equal rates across males and females (Strømme et al., 2002). People with Williams syndrome have distinct facial features, cardiovascular complications, and often co-occurring mild-to-moderate intellectual disability, attention problems, and anxiety (Mervis, 2000; Collins et al., 2010). Children with Williams syndrome also exhibit strong motivation for social interaction, indiscriminate friendliness, empathy for others (Järvinen et al., 2013), and relative strengths in verbal short-term memory and concrete expressive language (Mervis and John, 2010).

Anxiety is one of the most prevalent co-occurring conditions among people with Williams syndrome (Stinton et al., 2012; Royston et al., 2017; Ng-Cordell et al., 2018). A comprehensive examination of anxiety among children and adolescents ages 4–16 with Williams syndrome (*n* = 119) found that 53.8% met diagnostic criteria for specific phobia and 11.8% met criteria for generalized anxiety disorder (Leyfer et al., 2006). Furthermore, examination of the type of phobias experienced showed that loud noises were the most prevalent (27.7%) compared to common phobia types (e.g., situational, natural environment). Longitudinal research has provided evidence that anxiety and fears experienced by children and adolescents with Williams syndrome tend to persist over time, interfere with daily life, and may negatively impact socioemotional development, pointing to the need for interventions for children with Williams syndrome and co-occurring fears and anxiety (Einfeld et al., 1997, 1999, 2001; Woodruff-Borden et al., 2010).

Intervention studies with individuals with developmental and intellectual disabilities that specifically target anxiety, fears, or phobias are scant, with the majority of research conducted with autism spectrum disorder (ASD) populations (Kreslins et al., 2015; Ung et al., 2015). CBT interventions have demonstrated some effectiveness for individuals with higher functioning ASD (Ung et al., 2015), but few studies have tested the effectiveness of behavioral interventions on specific fears among people with ASD and co-occurring intellectual and developmental disabilities (Rosen et al., 2016). Single-subject design studies utilized systematic desensitization, graduated exposure, reinforcement procedures (some implemented by parents), modeling, hierarchy/stimulus fading, among other behavioral strategies (Rosen et al., 2016). Notably Koegel et al. (2004) conducted a within-subjects study targeting auditory-based fears (fear of toilet-flushing, animal toy sounds, and noises related to vacuum cleaners, blenders, and hand mixers) with three children with ASD and co-occurring developmental and/or intellectual disabilities. The results showed that all three children in this study were comfortable in the presence of their previously feared stimuli following the systematic desensitization intervention and at follow-up (based on child progress through their fear hierarchies) (Koegel et al., 2004). The treatment of anxiety and co-occurring intellectual disability is largely understudied. Case studies provide some evidence of effectiveness of graded exposure, response prevention, psychoeducation, and relaxation techniques to treat specific phobias in adults with intellectual disabilities (Hurley, 2004; Cowdrey and Walz, 2015; Dagnan et al., 2018). Further treatment effectiveness research is warranted to address the needs of individuals with intellectual disabilities and co-occurring anxiety.

Treatment studies with people with Williams syndrome and anxiety are exiguous, with the vast majority including case studies with small samples (1–3 participants) (Klein-Tasman and Albano, 2007; Phillips and Klein-Tasman, 2009; Conelea and Klein-Tasman, 2013). These case reports demonstrate some success treating anxiety symptoms using CBT-based interventions, with a tailored emphasis on behavioral aspects such as repeated practice of skills and role-playing replacement behaviors and less emphasis on cognitive components such as cognitive restructuring. Essau and Longhi (2013) found improvements in emotional and social skills with reduced anxiety following a case study analysis using a six-session CBT-based approach that taught skills connecting thoughts, emotions, and behaviors: Emotional and Social Skills Training for Individuals with Williams Syndrome (ESST-WS). Recent research of an adapted virtually delivered CBT-based group intervention for adults with Williams syndrome and anxiety (*n* = 4) showed promise as a feasible and effective approach (Thom et al., 2022). In sum, the research literature on evidence-based interventions with children and adults with Williams syndrome and co-occurring anxiety is remarkably scant, with few published studies, none with younger children, and none (outside very recent work by our group) specifically focused on fears and phobias.

In younger children with Williams syndrome, fears are a central manifestation of anxiety. While for typically developing children anxiety symptoms relating to specific phobias decrease over time with development (Costello et al., 2011), children with Williams syndrome and/or co-occurring intellectual disability are likely to experience worsening anxiety symptoms across development (Maiano et al., 2018; Ng-Cordell et al., 2018). Cognitive behavioral interventions have been identified as empirically supported approaches to addressing fears and phobias in typically developing children (Hirshfeld-Becker et al., 2010; Ollendick and Davis, 2013; Whiteside et al., 2020) and have also shown benefit for children with ASD (Davis et al., 2007; Ollendick et al., 2021) and children with intellectual disabilities (Hagopian et al., 2001; Moskowitz et al., 2019; Dovgan et al., 2020; Fodstad et al., 2021). Furthermore, evidence is accumulating that these interventions are also useful, with developmentally appropriate incorporation of play activities, with young typically developing children (Oar et al., 2015; Kershaw et al., 2017; Farrell et al., 2018). Leveraging the socially motivated tendencies of individuals with Williams syndrome (Mervis et al., 2001) by using social, adult–child play and humor in the context of a behavioral intervention may be useful to treat anxiety in children with Williams syndrome. We are engaged in a research program to examine the impact of play- and humor-infused exposure therapy on fears in children ages 4 through 10 with Williams syndrome and have described preliminary evidence of the utility of this approach (Klein-Tasman et al., 2022). The approach used is based in well-established graduated exposure (APA Division 12 Society of Clinical Psychology, 2022), uses functional behavior assessment to tailor treatment (Davis et al., 2009), and is informed by client-directed principles of outcome-informed therapy (Duncan and Miller, 2000). In our prior study, we demonstrated reductions in fear and anxiety (based on clinician and/or parent-report metrics) with four of the eight participants with complete data who participated in a brief, play- and humor-infused exposure-based intervention. Hence, this play- and humor-based behavioral approach shows promise as a developmentally attuned systematic desensitization intervention to treat fears and phobias in children with Williams syndrome. However, one significant limitation of the prior work was reliance on therapist and caregiver ratings of improvement rather than direct observation.

The current study employs behavioral coding to examine patterns of therapist use of graduated exposure and systematic desensitization using play and humor, and child anxiety behaviors in a subset of children with Williams syndrome who showed a positive response to the brief intervention in the pilot study (Klein-Tasman et al., 2022). For the purposes of this study, fear was operationally defined as observable child behaviors that indicated the child was experiencing emotional and/or physiological responses reflecting reluctance to engage with or an aversion to the stimulus. We hypothesized that children with Williams syndrome in this sample would exhibit observable reductions in fear within and across sessions, facilitated by this play- and humor-infused exposure therapy approach. We anticipated that the therapist techniques (use of play and humor during exposures, gradual exposure up the fear hierarchy by stimulus type (media, toy, and actual), and invited attention and passive exposure) would facilitate reduced anxiety and improved regulation, evidenced by increased frequency of tolerating and/or positive behaviors in the presence of the feared stimulus.

## Materials and methods

### Participants

Participants were recruited through the Williams Syndrome Association's research registry as well as referrals from the PI of a largescale study of the cognitive and behavioral phenotype in Williams syndrome. Participants included four children with Williams syndrome who initially had high levels of anxiety and then showed reduced fear and anxiety based on parent and/or clinician report in our preliminary work (Klein-Tasman et al., 2022). Participants range from 4 to 9 years of age and include three boys and 1 girl. See Table 1 for participant characteristics. Inclusion criteria included a diagnosis of Williams syndrome (confirmed through prior genetic testing), age 4–10, the presence of fear or a strong emotional response to a definable stimulus that could feasibly be addressed in a university-based research laboratory, and English as the primary language spoken.

**Table 1:** Participant characteristics.

**Participant pseudonym**	**Sex**	**Age (years; months)**	**GCA SS**	**Non-verbal reasoning SS**	**Verbal SS**	**ASD clinical diagnosis**	**Primary fear**	**Total sessions**	**Total sessions w/primary fear**	**Session length (range)**
Ashton	M	6;8	63	79	85	No	Hand Dryer	3	2	23–35 min
Beth	F	6;5	82	108	83	No	Blenders	3	3	45–55 min
Colton	M	4;1	63	97	59	Yes	Blood Pressure	4	4	25–30 min
Danny	M	9;2	69	66	85	No	Vacuum	4	3	40–45 min

GCA, general cognitive ability; non-verbal and verbal standard scores (SS) on the DAS-II.

### Measures

#### Background questionnaire

This parent-report questionnaire includes questions about child demographics, child's academic background, family information, birth and developmental history, medical history, and parent's primary concerns.

#### Differential Ability Scales – Second Edition

The Differential Ability Scales – Second Edition (DAS-II; Elliott, 2007) is a clinical measure of cognitive functioning for children ranging from age 2 years and 6 months to 17 years and 11 months. Children younger than age 9 were administered the Early Years battery, and children older than age 8 were administered the School Age battery. This instrument is used to identify where a child's abilities fall in comparison with their peers as well as an individual profile of their cognitive strengths and weakness. Scores are represented as standard scores. See Table 1 for participant scores.

### Procedure

#### The play- and humor-infused exposure-based intervention

The therapy approach is summarized as follows: The therapist provides a space for free play with toys that are tailored to the child's interests. The therapist encourages the parent(s) and caregiver(s) to participate and/or observe throughout sessions. After the child acclimates to the therapy room and happily plays together with the therapist, the therapist begins introducing the feared stimulus using the least intimidating level of exposure, which may be talking about the item, showing video of the item, or incorporating some aspect or representation of the item into the adult–child play (e.g., a pretend form of the item) and gauges the intensity of their reaction. As the child exhibits increased comfort in the presence of the feared stimulus, the therapist uses graduated exposure techniques to work up a fear ladder (based on functional assessment discussion with the parent prior to the intervention) toward direct contact with the feared stimulus. The therapist flexibly moves up and down levels of the fear hierarchy depending on the child's response following presentation or encouraged engagement with the feared stimulus. Throughout the sessions, socially engaging interactive play with the child is the consistent backdrop to help maintain contact and positive engagement with the feared stimulus. When anxiety becomes heightened as exposures incrementally move toward direct contact with a phobic stimulus, the therapist is likely to use socially attuned humor with the child (e.g., playful exaggerated emotion as a form of systematic desensitization) to help co-regulate, that is, to help the child sustain an emotionally regulated state, and to align themselves with the child, model emotional expression, and introduce a new emotional tone to interaction with the feared stimulus. In summary, the therapist spontaneously decreases the degree of exposure and/or increases playfulness and humor if the child shows signs of dysregulation and then increases the degree of exposure if the child appears regulated. Across sessions, the goal is for the child to sustain an emotionally regulated state, as manifested by exhibiting fewer fearful and anxious reactions to their feared stimulus to reduce functional impairment.

#### Behavioral coding procedure

Archival video-recorded intervention sessions of four participants (total of 12 sessions) were coded and analyzed using Noldus Observer XT 15.0 software to determine how child fear and anxiety-related behaviors change within session and across sessions, and characterize therapist use of play and humor and exposure techniques in this therapy. Behavioral coding (systematically defining and identifying overt behaviors) was conducted based on the coding scheme described in Tables 2, 3.

**Table 2:** Therapist behavior coding scheme.

**Behavior code (Duration)**	**Operational definition**
**Therapist exposure type**	
Pure exposure	The therapist presents the feared stimulus without play or humor (e.g., blood pressure cuff) with or without invited attention to the feared stimulus, or prompts the parent/ caregiver to present the child with the feared stimulus without play or humor.
Exposure with play and humor	The therapist presents the feared stimulus, with or without invited attention to the feared stimulus, with play and humor attempts to engage with the stimulus. This may be observed as a video playing in the background, and the therapist prompts play or humor (e.g., playful narrating techniques with humor) to engage with the media example. This may also be coded if the therapist prompts the parent/ caregiver to use play or humor with the child during exposure.
**Stimulus type**	
Media	Use of media version of feared stimulus in either pure exposure or exposure with play and humor. The therapist presents the child with a media example (image, video, or other media version) of the feared stimulus or the media example is present/ in view.
Toy	Use of a toy version of the feared stimulus in either pure exposure or exposure with play and humor. The therapist presents the child with a toy example (doll and toy vacuum cleaner) of the feared stimulus or the toy example is present/ in view.
Actual	Use of actual (realistic) version of feared stimulus in either pure exposure or exposure with play and humor. The therapist presents the child with their feared stimulus (e.g., blood pressure cuff) or the feared stimulus is present/ in view.
**Attention to feared stimulus**	
Invited attention	Therapist-initiated verbal or gestural cues or prompts attempting to direct the child's attention to the feared stimulus indicates presence of invited attention. Invited attention can also be observed in the form of therapist narration immediately before (primer) exposure, during exposure, or immediately after the exposure (within 3 seconds of exposure termination). Invited attention cannot occur during an independent child behavior.
Passive exposure	Invited attention toward another stimulus that is not the feared stimulus, thereby making the feared stimulus secondary to the invited attention stimulus.
**Parent exposure type**	
Pure exposure	The parent spontaneously presents the child with their feared stimulus, with or without invited attention to the exposure stimulus, without play or humor.
Exposure with play and humor	The parent spontaneously presents the feared stimulus, with or without invited attention to the exposure stimulus, with play and humor attempts to engage with the stimulus. This may be observed as a video playing in the background, and the parent prompts play or humor (e.g., playful narrating techniques with humor) to engage with the media example.

**Table 3:** Child behavior coding scheme.

**Behavior code**	**Operational definition**
**Negative behaviors**	
Negative verbalizations	Child verbally protests the interaction with the feared stimulus (utterance) or otherwise makes sounds indicating discomfort during exposure, such as saying “No!”, “I don't like that”,etc.(vocalization).
Negative physical	Child displays a negative physical reaction to the feared stimulus, such as cowering, backing up, or placing their hands over their ears (i.e.,avoidance).
**Neutral behaviors**	
Tolerating presence of feared stimulus	Child does not display any observable positive or negative reaction to the exposure (feared stimulus), indicating tolerance of the feared stimulus.
**Positive behaviors**	
Positive verbalizations	Child verbally exhibits excitement or engagement with the feared stimulus through articulated speech (utterance) or produces verbal indication (vocalization), grunt, or other verbal approximation indicating excitement or enjoyment during exposure, such as saying, “I want to try”, “wow!”, etc.
Positive physical	Child displays a positive physical reaction to the feared stimulus, such as moving their body toward the feared stimulus or engaging in exposure with physical contact, which followed a therapist/parent/caregiver prompt or cue.

Therapist behaviors were coded using duration and frequency recording. The duration of therapist use of play- and humor-infused exposure therapy and pure exposure was coded to identify patterns of therapist-led exposure by type, as well as spontaneous parent use of exposure by type. The use of different types of feared stimuli (media, toy, and actual) was also coded during pure or play- and humor-infused exposure therapy. Therapist behaviors also included passive and invited attention to the feared stimulus. For example, the therapist may direct attention to a non-feared stimulus (e.g., toy car), thereby making the feared stimulus secondary to the stimulus where attention is invited (i.e., passive exposure). If the therapist used verbal and/or non-verbal cues to guide the child's gaze toward the feared stimulus (e.g., “Hey want to check this out?”), this was coded as invited attention to the stimulus. See Table 2 for the detailed therapist behavior coding scheme.

Child behaviors were coded as frequency of positive, tolerating (i.e., neutral), and negative behaviors within intervals, to capture observable fear-related behaviors. Verbalizations (e.g., articulated speech or verbal approximations) and physical behaviors (e.g., physical approach or avoidance) were also coded. See Table 3 for the detailed child behavior coding scheme.

### Data analytic approach

#### Interrater reliability process

Interrater reliability was calculated using Noldus Observer XT 15.0, including Cohen's kappa and agreement percentage. Cohen's kappa ranges from −1 to +1, and kappa values should be interpreted using the following ranges: Values of ≤ 0 suggest no interrater agreement, values of 0.01–0.20 suggest no agreement to slight agreement, values of 0.21–0.40 suggest fair agreement, values of 0.41–0.60 suggest moderate agreement, values of 0.61–0.80 suggest substantial agreement, and values of 0.81–1.00 suggest almost perfect interrater agreement. For the current study, interrater reliability was considered met when k ≥0.70 and agreement percentage ≥80%.

The first author and a graduate student trained in the coding scheme separately coded one intervention session video (of a participant that is not included in the current sample) to pilot the developed coding scheme. It was expected that the coding scheme would need revisions as the development of a behavior coding scheme is an iterative process, often requiring discussions about revisions and refinement of operational definitions (Chorney et al., 2015). Macrocoding (i.e., codes that may apply to the broader context of the behaviors) and microcoding approaches (i.e., codes that are more specific and time-intensive) to the coding scheme were considered during the revision process (Chorney et al., 2015). The two coders discussed areas of disagreement during the preliminary coding process and revised the coding scheme accordingly. When all child codes and all therapist codes were analyzed as respective groups, minimum kappa values were reached. For all child codes, agreement percentage for frequency recording was 87.90%, and k = 0.84. For all therapist codes, agreement percentage for frequency was 82.46%, and k = 0.79 and agreement percentage for duration was 92.69%, and k = 0.91. See Table 4 for detailed reliability coding.

**Table 4:** Interrater reliability.

**Code**	**Preliminary reliability**	**Preliminary reliability**	**Ashton**		**Beth**		**Colton**		**Danny**	
	**Frequency**	**Duration** ^*^	**Frequency**	**Duration** ^*^	**Frequency**	**Duration** ^*^	**Frequency**	**Duration** ^*^	**Frequency**	**Duration** ^*^
*Negative child behaviors*	88.89 %	89.10%	80%	72.67%	92.86%	94.78%	85.71%	60.44%	NP	NP
Negative vocalizations	80%	96.61%	100%	55.58%	NP	NP	NP	NP	NP	NP
Negative utterances	100%	90.27%	0% ^**^	0% ^**^	80%	45.66%	100%	53.57%	NP	NP
Negative physical	84.62%	87.71%	100%	77.68%	95.65%	96.88%	83.33%	61.05%	NP	NP
*Positive child behaviors*	89.74%	94.06%	86.67%	56.67%	88.89%	74.19%	86.49%	90.15%	88.89%	75.16%
Positive vocalizations	88.89%	87.51%	100%	59.63%	NP	NP	83.33%	56.91%	NP	NP
Positive utterances	84.62%	89.38%	83.33%	95.71%	95.45%	90.87%	NP	NP	100%	91.67%
Positive physical	94.12%	98.56%	83.33%	37.38%	82.61%	71.75%	88.00%	94.22%	87.50%	74.56%
*Neutral/Tolerating child behaviors*	86.21%	65.26%	91.67%	86.18%	92.31%	79.21%	86.67%	80.57%	NP	NP
**All child codes**	**87.90%**	**71.78%**	**87.23%**	**65.41%**	**90.70%**	**81.44%**	**86.44%**	**83.79%**	**88.89%**	**75.16%**
Therapist – pure exposure	92.86%	84.45%	NP	NP	100%	95.51%	NP	NP	NP	NP
Therapist – play and humor	83.33%	98.05%	100%	99.15%	100%	95.25%	75.00%	100%	100%	88.56%
Therapist – stimulus type: media	89.47%	97.66%	NP	NP	100%	95.56%	NP	NP	NP	NP
Therapist – stimulus type: toy	50%	94.52%	50%	99.48%	100%	81.75%	100%	80.79%	100%	89.42%
Therapist – stimulus type: actual	66.67%	89.06%	NP	NP	66.67%	83.30%	66.67%	94.74%	100%	95.29%
Therapist – directed attention	88.41%	92.67%	72.22%	85.23%	89.58%	96.03%	94.44%	82.97%	61.54%	92.29%
Therapist – passive exposure	100%	86.51%	NP	NP	NP	NP	66.67%	85.28%	NP	NP
Parent – pure exposure	81.25%	95.23%	NP	NP	NP	NP	NP	NP	NP	NP
Parent – play and humor	N/A	N/A	NP	NP	NP	NP	100%	93.17%	NP	NP
**All therapist codes**	**82.46%**	**92.69%**	**70.83%**	**95.70%**	**91.78%**	**91.14%**	**80.43%**	**93.54%**	**68.75%**	**91.10%**

Interrater reliability values are reflected as percent agreement (%).

^*^Duration interrater reliability values are conservative, reflecting agreement on milliseconds(ms).

^**^Negative utterances disagreement was a disagreement of 1 observation (frequency) and 1 second(duration).

N/A: not applicable to the videocoded.

NP: behavior code not present in this session video.

Once interrater agreement of the behavior coding scheme was established (agreement ≥80% and/or k ≥0.70) with one intervention session video of a participant that is not included in the current sample, one randomly selected intervention video for each participant was coded to ensure continued interrater reliability for all participants in this sample. Once interrater reliability was established with four intervention session videos (one for each participant), series data trends of frequency and duration of behaviors (per session and across) were coded, graphed, and visually inspected. Based on the interrater reliability findings, we elected to concentrate on frequency of child behavior and duration and frequency of therapist behavior. Specifically, the number of child behavior occurrences in each session of each participant (frequency) was calculated within 5-minute intervals and graphed and visually inspected to demonstrate patterns of change. Similarly, the duration and frequency of therapist behaviors (e.g., use of play and humor) in each session were coded and calculated within 5-minute intervals and graphed and visually inspected. Lag sequential analyses (event state lag of 1) were also conducted to identify sequential patterns between therapist and child behaviors as well as sequential therapist behaviors (e.g., invited attention followed by exposure with play and humor).

## Results

### Child demographics and overview of course of treatment

Each of the four participants' demographic information as well as verbal and non-verbal norm-referenced cognitive functioning are displayed in Table 1. The fear hierarchy (i.e., course of treatment) for each participant is displayed in Table 5.

**Table 5:** Description of fear hierarchies.

**Participant pseudonym**	**Targeted fear**	**Fear hierarchy**
Ashton	Hand dryers	1. Watched videos of a variety of hand dryers in action, with increasing volume levels 2. Used portable hand dryer in the therapy room to blow scarves, push toy school bus first with the therapist placing the objects 3. Over time used portable hand dryer in the therapy room to blow scarves, push toy school bus with the child placing objects 4. Visited hand dryers in nearby restrooms, taking along scarves to play with, and therapist facilitates exposures 5. Finally, used hand dryer in naturalistic setting (i.e., public restroom) on hands, and child initiating exposures
Beth	Blenders	1. Played with toy blender with therapist initiation 2. Played with toy blender without therapist initiation 3. Watched videos of varying blenders with increasing volume 4. Therapist placed actual blender in the therapy room (unplugged) in the child's view 5. Therapist activated actual blender in the therapy room (without content) for short durations, with child observing/listening from next door and eventually entering the room 6. Therapist activated actual blender in the therapy room (without content) for increasingly longer durations, with child observing/listening from next door and eventually entering the room 7. Therapist activated actual blender in the therapy room with ice/berries, with child observing from next door and eventually entering the room 8. Therapist activated actual blender while child was in the room 9. Therapist activated actual blender while child was in the room, with child in closer proximity to the blender (< 3′) 10. Therapist activated actual blender while child added berries 11. Child activated the blender
Colton	Blood pressure (BP) cuff	1. Therapist and child played with toy BP cuff on stuffed animal, with therapist initiating 2. Therapist and child played with real BP cuff on stuffed animal, with therapist initiating 3. Therapist and child played with toy BP cuff on stuffed animal, with child initiating 4. Therapist initiated play with toy BP cuff on therapist (e.g., arms and legs) 5. Child initiated play with toy BP cuff on therapist (e.g., arms and legs) 6. Therapist initiated play with real BP cuff on therapist 7. Child initiated or joined play with real BP cuff on therapist 8. Therapist initiated play with toy BP cuff on child 9. Therapist initiated play with real BP cuff on child
Danny	Vacuum cleaners	1. Therapist initiated play with toy vacuum cleaner without sound 2. Child initiated play with toy vacuum cleaner with sound 3. Watched videos of varying vacuum cleaners with increasing volume 4. Therapist presented (actual) vacuum cleaner in the room, unplugged 5. Therapist presented (actual) vacuum cleaner in the room plugged in but turned off. 6. Therapist initiated play with (actual) vacuum cleaner (turned on) in the therapy room with the child in a separate room 7. Therapist initiated play with (actual) vacuum cleaner (turned on) in the hallway with the child seated on rolling chair and gradually brought closer (with child's permission) by throwing a stuffed animal closer and closer toward the vacuum cleaner 8. Therapist playful use of (actual) vacuum cleaner (modeling) to “vacuum” up bubbles 9. Therapist initialed playful use of (actual) vacuum cleaner by the child to “vacuum” up bubbles 10. Child initiated use of (actual) vacuum cleaner (by the child) to vacuum debris from the carpet

### Behavioral coding analyses

The duration of each therapist behavior code was analyzed using duration-within-interval recording. Intervals were also 5-minute each across the duration of each session.

The frequency of each child behavior code was calculated, graphed, and visually inspected using frequency-within-interval recording. Intervals were defined as 5-minute each across the duration of each session. Each child participated in 3–4 therapy sessions. For two participants, one session video was excluded because the intervention focus was on a secondary feared stimuli (i.e., not the primary targeted fear identified). Each session video duration ranged from 30 to 65 min as indicated in Table 1.

Analyses of temporal patterns were conducted using lag sequential analyses in Noldus Observer XT 15.0. Lag sequential data are defined as identifying an event that immediately follows an event (i.e., state lag event of 1). Lag sequential data for therapist behavior codes following child behavior codes and child behavior codes following therapist behaviors were analyzed to identify high frequencies of sequential behavior patterns. First, lag sequential data for therapist behaviors following child behavior codes were analyzed to identify ways in which the therapist responded to the child, within and across sessions. Second, lag sequential data for child behaviors following therapist behaviors were analyzed to identify how each child responded to each therapist technique within the context of exposure therapy.

Each participant's behavioral coding data analyses will be presented individually, using pseudonyms to protect privacy for each child, in a case series design format. Of note, passive exposure was only occasionally observed and is therefore not interpreted here. To illustrate the unique response to the intervention observed for the different participants in this sample, we have individually tailored presentations of the findings to best capture the nature of each participant's response. Common behavioral coding trends across participants will be presented in the discussion.

#### Ashton

Ashton's primary targeted fear was hand dryers found in public restrooms. Therapy sessions 1 and 3 were coded and analyzed as these sessions focused on this primary feared stimulus. Below child behavior (frequency coding) will be discussed first, followed by therapist behaviors (duration coding) and last, lag sequential findings (state event lag of 1).

**Figure 1:**
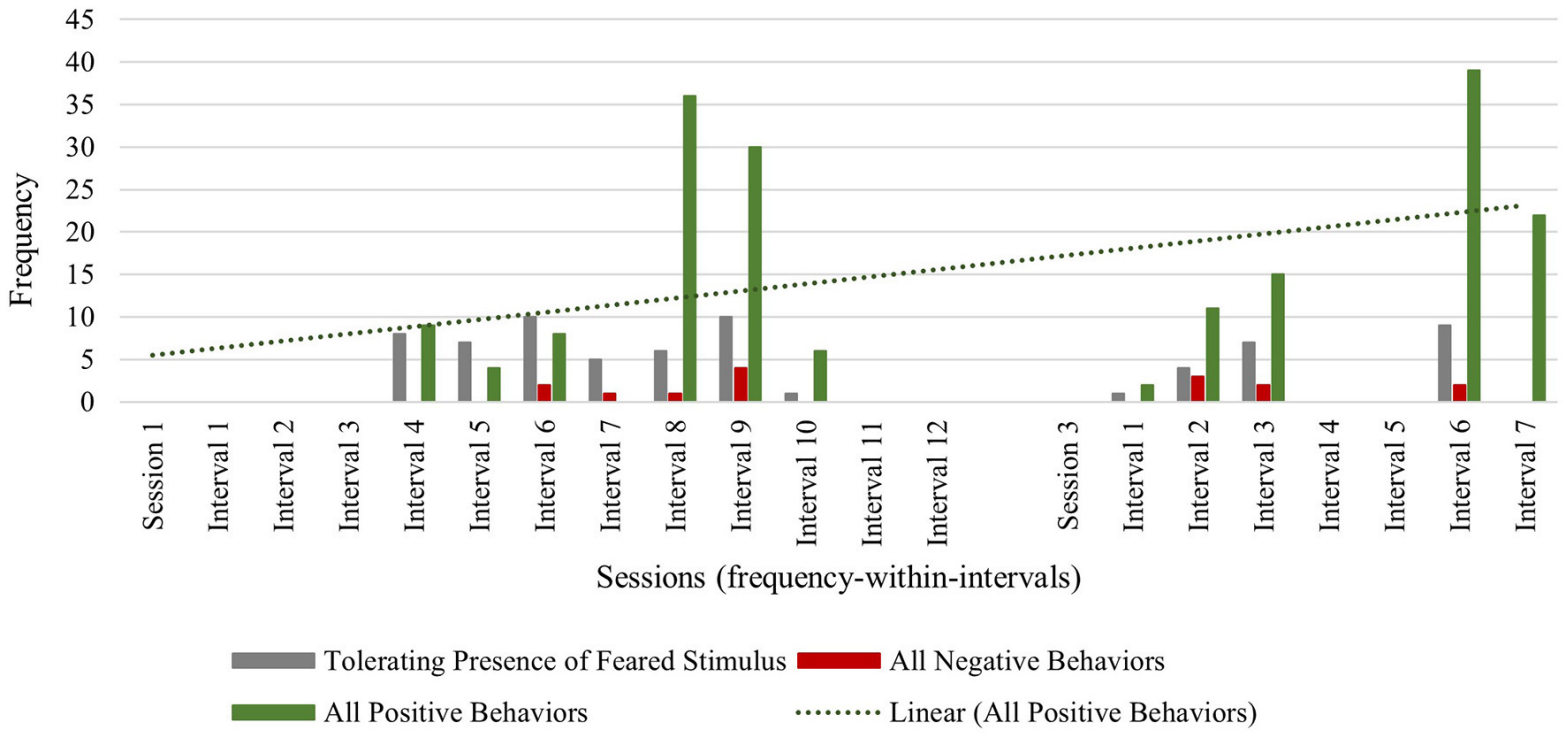
Ashton: across session child behavior.

Across sessions, Ashton exhibited low frequencies of all negative behaviors (verbalizations and physical avoidance), with a total frequency of 8 in session 1 and a total frequency of 7 in session 3. This highlights the flexible and attuned nature of the intervention, where the therapist works to minimize overly distressing the child during exposures. The observed frequency of tolerating the feared stimulus decreased from a total frequency of 47 in session 1, to a total session frequency of 21 in session 3. However, this decrease in tolerance of the feared stimulus can be explained by the maintained frequency of positive verbalizations (frequency of 34 during both sessions 1 and 3) and even an increase in positive physical behaviors (e.g., approach or direct contact with the feared stimulus) from session 1 (frequency of 33) to session 3 (frequency of 35) (see Figure 1).

**Figure 2:**
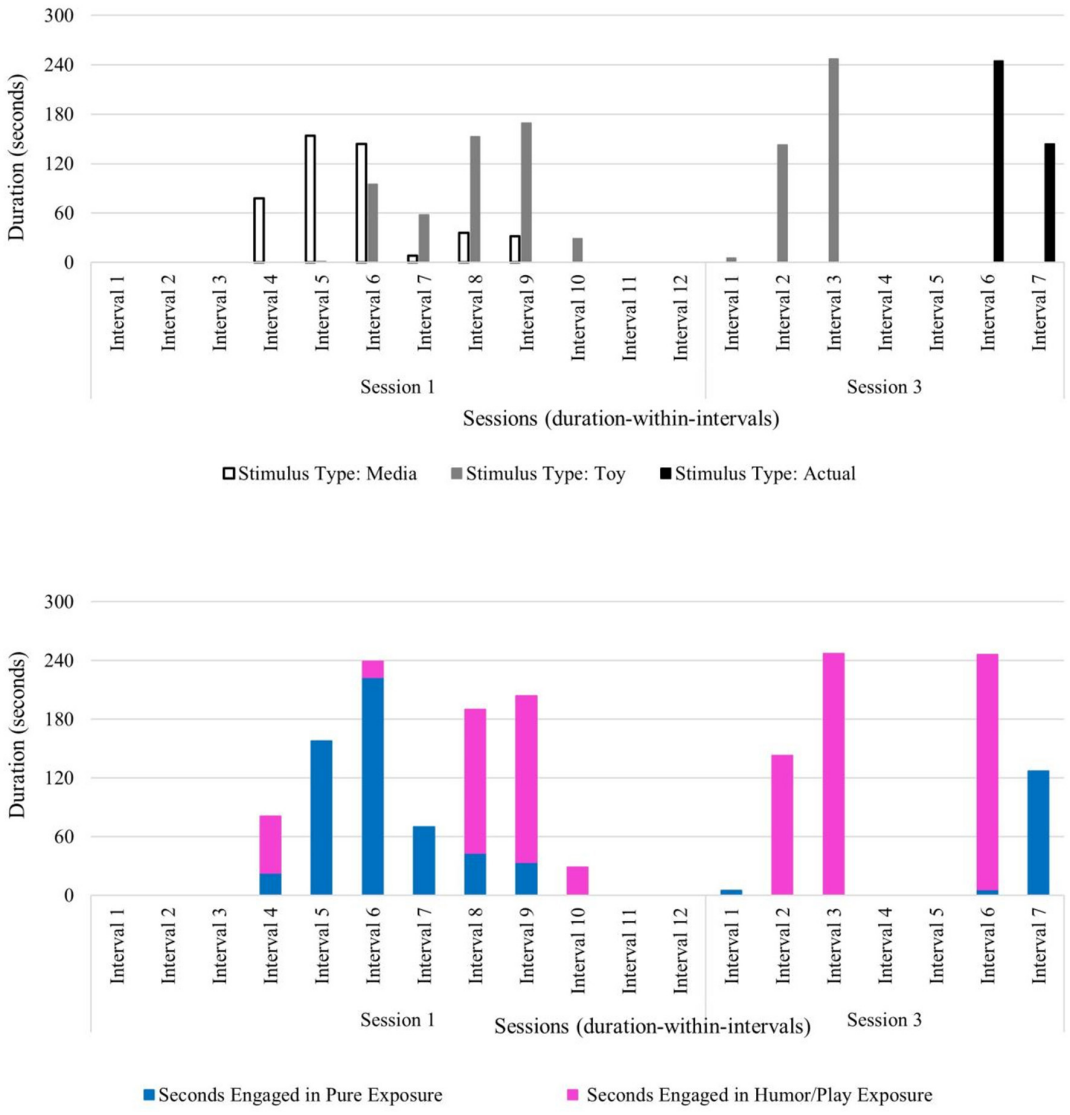
Ashton: therapist stimulus type and exposure type across sessions.

Furthermore, Ashton's observed positive behaviors maintained and increased in the face of increasingly challenging stimulus types used by the therapist across sessions. In session 1, the therapist used pure exposure with media stimulus types (i.e., least challenging stimulus type), followed by exposure with play and humor using a toy stimulus type (i.e., a portable hand dryer) (see Figure 2). In the first half of session 3, the use of play and humor was paired with the toy version of the hand dryer along with allowing scarves to be blown by the hand dryer, to engage with the stimulus in a new and unconventional way. In the latter half of session 3, this child engaged in increasingly longer durations of pure exposure with the most challenging stimulus type: an actual hand dryer in a community restroom (see Figure 2). The most frequent child behavior group in session 3 was positive behavior (frequency of 89), relative to tolerant behavior (frequency of 21) and negative behavior (frequency of 7). Across sessions, Ashton's progress was observed through the frequency of positive verbalizations, and an increase in positive physical behaviors (e.g., approach or direct contact with the feared stimulus) in the presence of his feared stimulus. These data reveal that over the course of this brief play- and humor-infused exposure therapy, this child was able to not only tolerate the feared stimulus, but also engage with the previously feared stimulus in positive ways with no observable signs of distress—even when faced with the most challenging level of his fear hierarchy (i.e., using an activated hand dryer in a community restroom) (see Figure 2).

Next, temporal patterns between child and therapist behavior codes were analyzed using lag sequential data analyses. First, temporal patterns between therapist use of invited attention and all child behaviors were analyzed. The results show that therapist use of invited attention most frequently followed the child tolerating the feared stimulus and also followed positive child behaviors and was less common following negative child behaviors. This differential use of invited attention demonstrates how the therapist uniquely tailors the timing of such techniques (e.g., redirection, narration, and exaggerated emotion to facilitate co-regulation). When a child is observed to tolerate a feared stimulus, it can appear quite ambiguous to the therapist. For instance, the child's absence of negative or positive behaviors can suggest to the therapist that a redirection to the exposure activity may be warranted to prevent an occurrence of negative behavior (i.e., proactive strategy) and to continue tolerance of the feared stimulus. The use of invited attention following the child's positive behaviors suggests that the therapist uses techniques to encourage continued positive engagement with the feared stimulus (see **Table 7**). Importantly, the results also show that following the therapist's use of invited attention, the most frequent child behavior code included positive physical behaviors (e.g., approaching or making direct contact with the hand dryer) (see Table 6). This pattern is suggestive that the therapist's technique to gently entice the child to participate in play-based exposures most frequently yields positive child behavioral responses.

**Table 6:** Frequency of child behaviors immediately following therapist use of invited attention.

**Session**	**Negative response**				**Tolerance of the feared stimulus**				**Positive response**			
	**Ashton**	**Beth**	**Colton**	**Danny**	**Ashton**	**Beth**	**Colton**	**Danny**	**Ashton**	**Beth**	**Colton**	**Danny**
Session 1	0	**13**	5	7	0	4	4	7	**10**	**19**	5	**12**
Session 2	N/A	**20**	0	4	N/A	4	4	4	N/A	**12**	9	**16**
Session 3	1	4	3	4	2	2	5	7	**19**	7	**18**	**19**
Session 4			7				6				7	

N/A, session was not coded for this participant.

Lag sequential frequencies of 10 or more are bolded.

#### Beth

Beth's primary targeted fear was identified as blenders. Therapy sessions 1, 2, and 3 were coded and analyzed as all sessions focused on this primary feared stimulus. Below child behavior (frequency coding) will be discussed first, followed by therapist behaviors (duration coding) and last, lag sequential findings (state event lag of 1).

**Figure 3:**
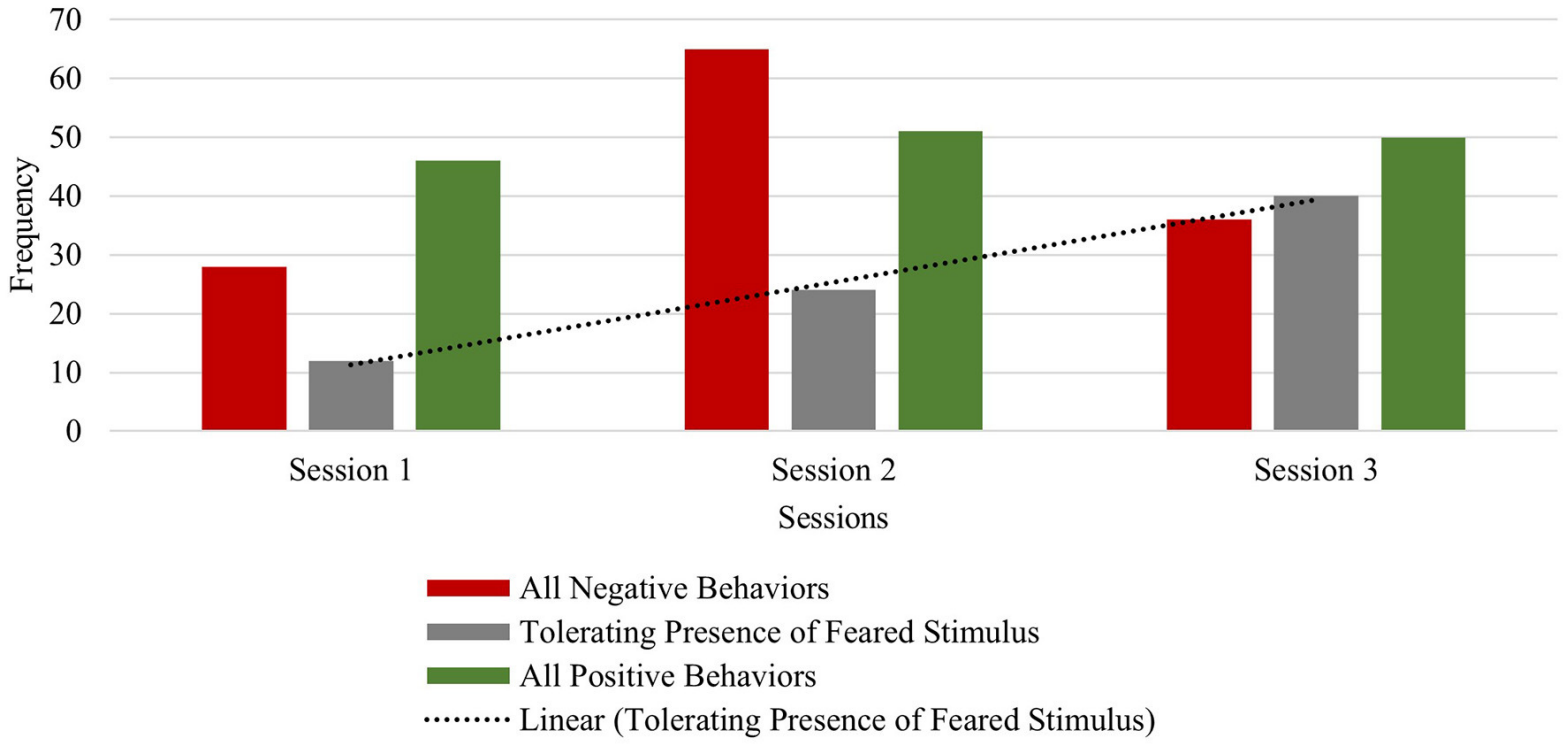
Beth: across session child behavior.

**Figure 4:**
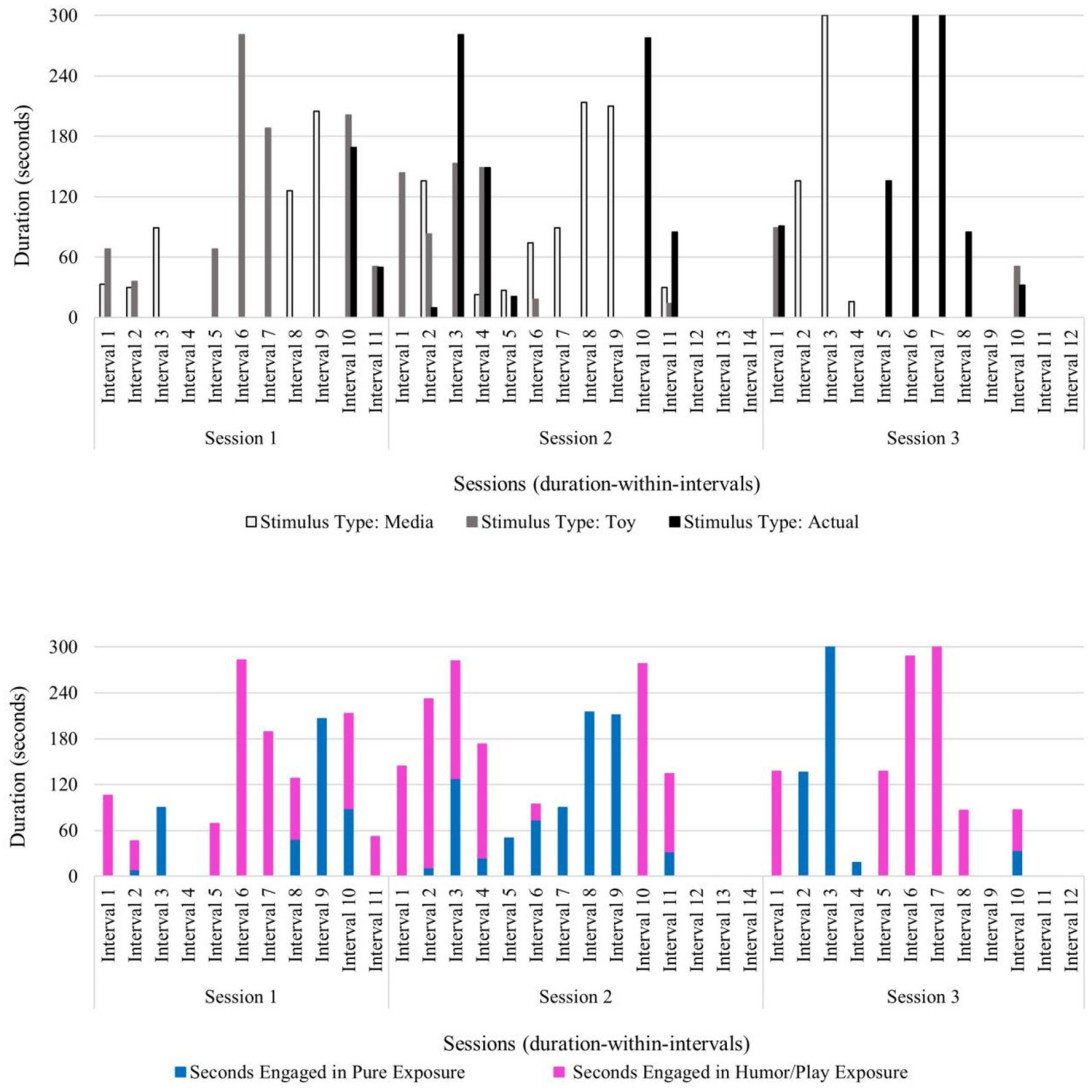
Beth: therapist stimulus type and exposure type across sessions.

Beth exhibited lower frequencies of within-session negative behaviors (verbalizations and physical avoidance) relative to within-session positive behaviors in the first session (negative behavior frequency of 28 and positive behavior frequency of 46) and in the final session (negative behavior frequency of 36 and positive behavior frequency of 50). Across session data show increasing frequencies of both negative and positive behaviors. It was observed that negative physical behaviors (e.g., covering ears) often co-occurred with positive physical behaviors (e.g., approaching the feared stimulus) within each of the three sessions. This behavior pattern has been highlighted in the literature as unique among children with Williams syndrome who experience fears and phobias (Gallo et al., 2008). Her verbalizations (both negative and positive) increased from session 1 (negative verbalizations: frequency of 4; positive verbalizations: frequency of 21) to session 3 (negative protest: frequency of 17; positive verbalizations: frequency of 34), while her physical behaviors decreased from session 1 (negative: frequency of 24; positive: frequency of 25) to session 3 (negative: frequency of 19; positive: frequency of 16). This increase in verbal protesting may be suggestive of attempted avoidance (similar to an extinction burst) of the feared stimulus as increasingly challenging levels of the fear hierarchy are presented over the course of this brief intervention (i.e., media to toy to actual blender) (see Figures 3, 4). Beth did exhibit some negative verbalizations while she simultaneously engaged in positive physical behaviors (e.g., approaching the blender). Notably, the observed frequency of Beth tolerating the feared stimulus showed a consistent increase across all sessions (frequency of 12 in session 1; frequency of 24 in session 2; frequency of 40 in session 3), with Beth making direct contact and operating an activated blender by session 3 (see Figure 3).

Beth's pattern of co-occurring negative and positive behaviors along with a steady increase in tolerating the feared stimulus likely resulted from the therapist's deliberate use of specific exposure type(s) paired with specific stimulus type(s) across sessions. The therapist used play and humor to co-regulate, to help Beth sustain emotional regulation, and to prevent anxiety from becoming overwhelming. In session 1, the therapist infused play and humor into exposures with all stimulus types (i.e., media, toy, and actual blender) and implemented pure exposure with media examples of blenders and brief durations of the actual stimulus. The actual blender was introduced near the end of session 1 and unplugged and turned off to begin exposure to the sight of the object before working up the fear hierarchy to auditory exposure of the blender turned on later in the course of treatment (see Figure 4).

Next, temporal patterns between child and therapist behavior codes were analyzed using lag sequential data analyses. First, temporal patterns between therapist use of invited attention and all child behaviors were analyzed. Findings reveal that during sessions 1 and 2, when the actual stimulus was presented to Beth and elicited some avoidance (i.e., negative behaviors) as well as interest in the blender (i.e., positive behaviors), therapist invited attention often followed all child behaviors (negative, tolerant, and positive) (see Table 7). Differential use of invited attention was often tailored to the type of child behavior exhibited. Narration, exaggerated emotion using toys with play and humor, and redirection prompts were often used in response to Beth's simultaneous negative (e.g., verbal protest) and positive behavior (e.g., direct contact with the blender) to re-engage or maintain participation in the exposures. Additionally, invited attention was also used as a priming technique to cue the child that an exposure activity was beginning to minimize upset or startle response with the transition. From sessions 1 and 2 to session 3, fewer negative child behaviors followed therapist invited attention. Rather, positive behaviors more often followed invited attention in session 3, suggesting effectiveness of the co-regulation efforts and encouraging the child to continue engagement in the therapy session—even as the stimulus type became increasingly challenging over the course of intervention sessions (see Table 6).

**Table 7:** Frequency of therapist invited attention immediately following child behaviors.

**Session**	**Negative behavior**				**Tolerance of the feared stimulus**				**Positive behavior**			
	**Ashton**	**Beth**	**Colton**	**Danny**	**Ashton**	**Beth**	**Colton**	**Danny**	**Ashton**	**Beth**	**Colton**	**Danny**
Session 1	0	**14**	2	9	**12**	7	6	**10**	**11**	**13**	7	6
Session 2	N/A	**27**	0	8	N/A	5	5	6	N/A	**13**	9	**12**
Session 3	1	9	4	6	6	5	8	7	**16**	4	**14**	**15**
Session 4	-	-	8		-	-	5		-	-	8	

N/A, session was not coded for this participant.

Lag sequential frequencies of 10 or more are bolded.

#### Colton

Colton's primary targeted fear was identified as blood pressure cuffs. Therapy sessions 1, 2, 3, and 4 were coded and analyzed as all sessions focused on this primary feared stimulus. Below child behavior (frequency coding) will be discussed first, followed by therapist behaviors (duration coding) and last, lag sequential findings (state event lag of 1).

**Figure 5:**
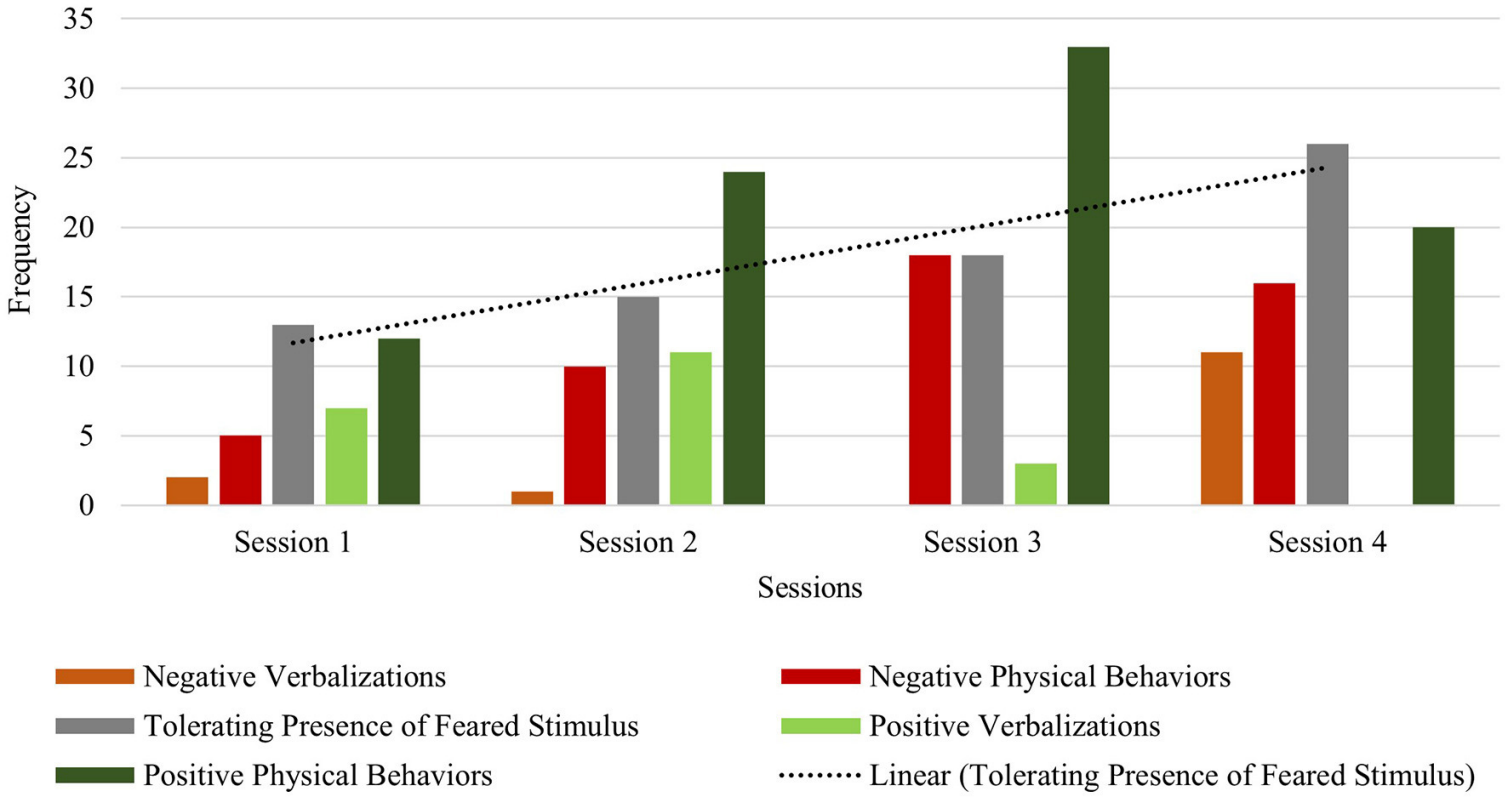
Colton: across session child behavior.

Colton exhibited lower frequencies of within-session negative behaviors (verbalizations and physical avoidance) relative to within-session positive behaviors in sessions 1 and 2. In session 3, frequencies of negative behaviors were equal to his tolerating behaviors (frequency: 18), with twice the frequencies of positive behaviors (frequency: 36) in response to his feared stimulus. In session 4, although negative behaviors increased, Colton's observed tolerating of the feared stimulus increased as well (negative behaviors: frequency of 27; tolerating behavior: frequency of 26) (see Figure 5). Colton exhibited a relatively lower frequency of within-session negative verbalizations compared to positive verbalizations in sessions 1–3. In session 4, however, the frequency of his within-session positive verbalizations was relatively lower than his within-session negative verbalizations. Notably, Colton demonstrated a steady across-session increase of tolerating his feared stimulus (session one frequency: 13; session two frequency: 15; session three frequency: 18; session four frequency: 26). Furthermore, positive physical behaviors increased across sessions from the first (frequency of 12) to the final session (frequency of 20), indicating more frequent approach and direct contact with blood pressure cuffs, which most often included the actual stimulus type in session 4. Taken together, Colton's improved tolerance and positive physical behaviors are suggestive of improved regulation skills in response to his previously feared stimulus over the course of this brief therapy approach (Figure 5).

**Figure 6:**
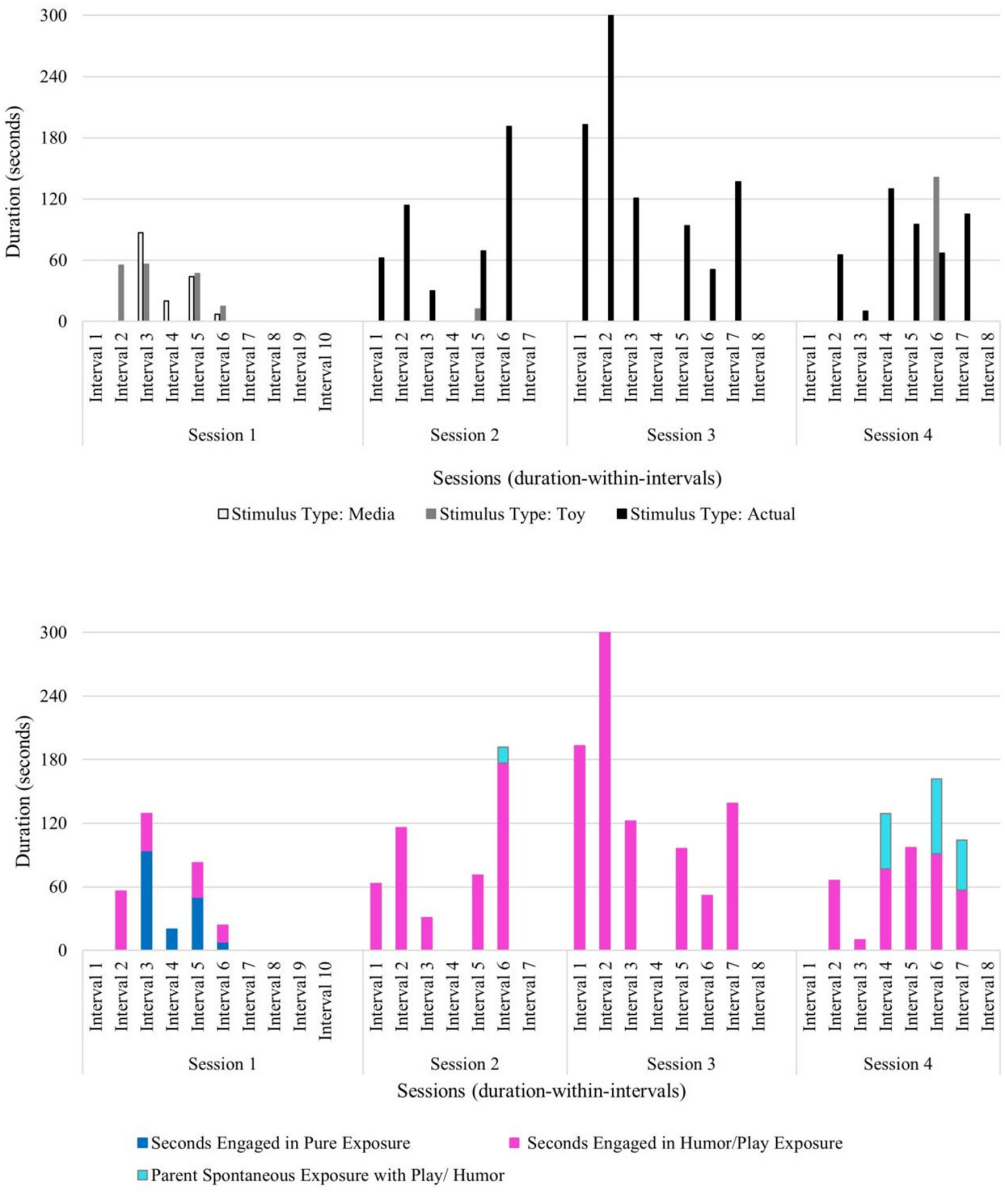
Colton: therapist stimulus type and exposure type across sessions.

The therapist attunement to Colton throughout treatment fostered the observed increase in his ability to tolerate his feared stimulus. Therapist use of pure exposure was brief and only used in session 1 with media examples of the feared stimulus. However, exposure with play and humor was used across all sessions with a toy version and the actual stimulus type (see Figure 6). Parent use of spontaneous play- and humor-infused exposure was also observed in session 2 (duration: 15 s) and session 4 (duration: 2 min, 50 s), suggesting that Colton's parent began acquiring and applying play- and humor-related skills with increasingly longer durations as therapy progressed (see Figure 6). The observed behavior pattern in Colton (who has relatively less developed verbal abilities compared to other children in this sample) highlights the benefit of this developmentally attuned intervention which incorporates adaptations to meet and respond to the individual child's needs and abilities during exposures. Colton's verbal score on the DAS-II was in the lower range (SS = 59), which speaks to the need for the therapist to gauge his behavior and non-verbal cues during exposures to sustain emotional regulation, avoid distress and upset, and minimize anxiety as they work up his fear hierarchy to the most challenging stimulus type: an actual blood pressure cuff. Taken together, the primary use of play and humor by the therapist and parent, along with shorter session durations, reflects the attunement to this child's individual needs.

Next, temporal patterns between child and therapist behavior codes were analyzed using lag sequential data analyses. First, temporal patterns between therapist use of invited attention and all child behaviors were analyzed. The results show that in response to varied child behavior (negative, tolerant and positive), the therapist frequently used invited attention techniques to either redirect the child to the activity (if negative child behavior was observed), use gesture or verbal prompts to gauge child's anxiety level (if child tolerating was observed), or narration in a playful and humorous way to continue child engagement in exposures (if positive child behavior was observed) (see Table 7). Following therapist invited attention, the most frequent observed child responses across sessions were positive physical behaviors (e.g., approaching the feared stimulus) and tolerance of the feared stimulus (i.e., blood pressure cuff) (see Table 6). These data provide some support for the effectiveness of the therapist's varied and tailored use of invited attention techniques with Colton considering he had limited verbal skills and was younger than the other participants.

#### Danny

Danny's primary targeted fear was identified as vacuum cleaners. Therapy sessions 1, 2, and 3 were coded and analyzed, but session 4 was not coded because the primary fear was not targeted during this session. Below, child behavior (frequency coding) will be discussed first, followed by therapist behaviors (duration coding) and last, lag sequential findings (state event lag of 1).

**Figure 7:**
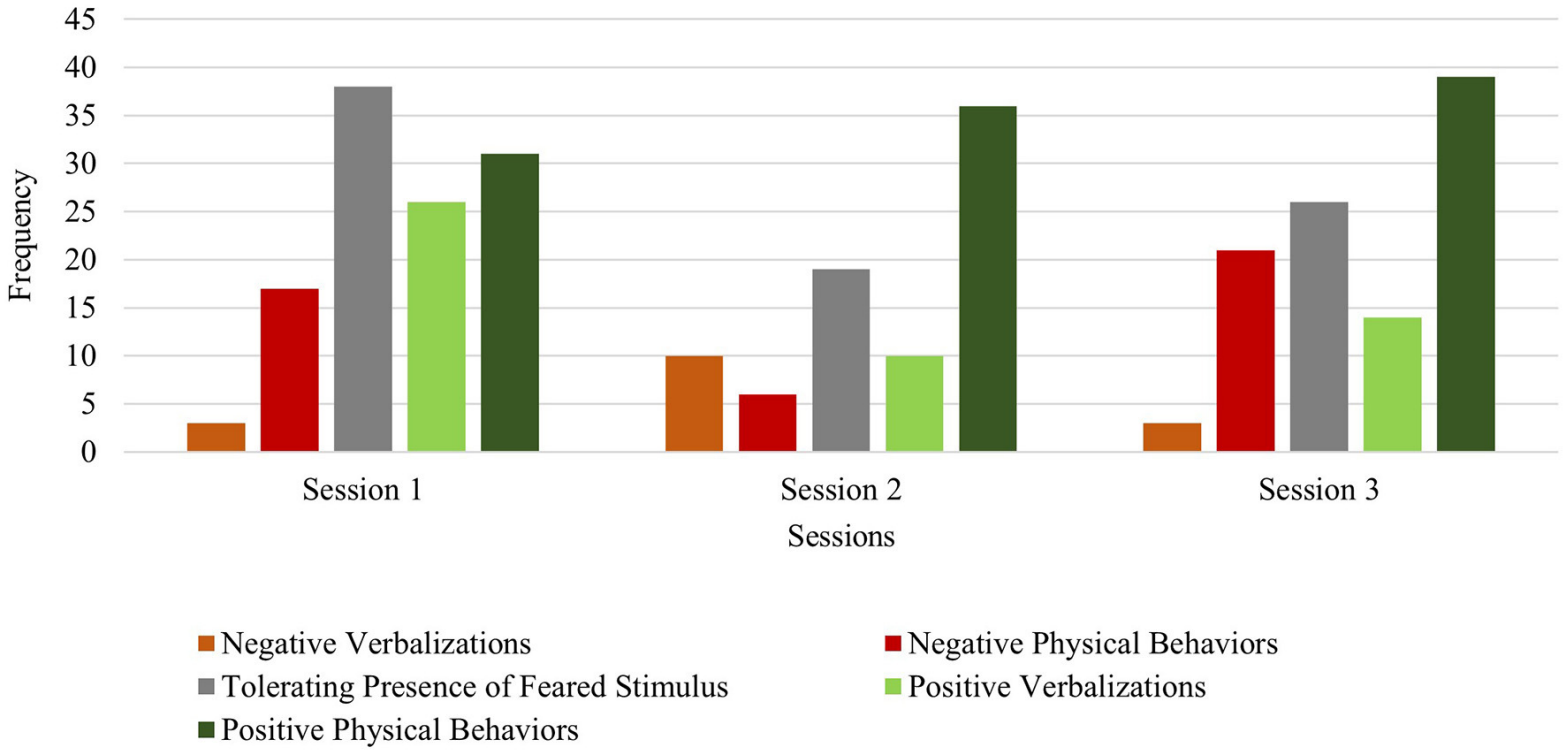
Danny: across session child behavior.

Danny exhibited lower frequencies of within-session negative behaviors (verbalizations and physical avoidance) relative to within-session tolerating and positive behaviors in session 1 (negative behavior frequency of 20; tolerating behavior frequency of 38; positive behavior frequency of 57), session 2 (negative behavior frequency of 16; tolerating behavior frequency of 19; positive behavior frequency of 46), and session 3 (negative behavior frequency of 24; tolerating behavior frequency of 26; positive behavior frequency of 53). Across session data show a slight increase in positive physical behaviors (i.e., approaching or making direct physical contact with the feared stimulus) from session 1 (frequency: 31) to session 3 (frequency: 39). Danny's profile of behavioral data trends shows his ability to tolerate and positively engage with vacuum cleaners within each session, even as the stimulus type became increasingly challenging (i.e., media to toy version to actual feared stimulus). It is notable that the child spent several minutes independently vacuuming the carpet in session 3 (see Figure 7).

**Figure 8:**
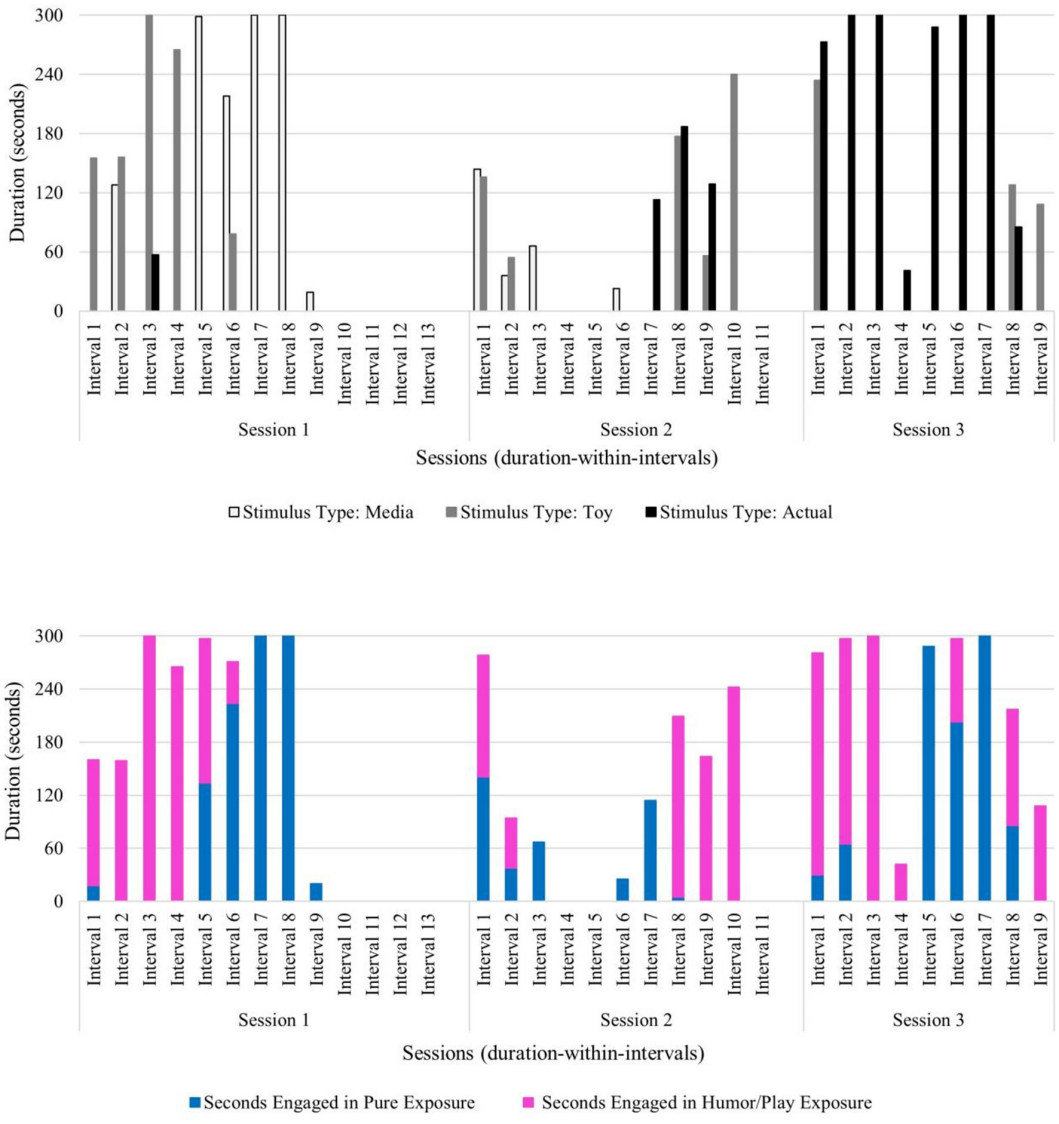
Danny: therapist stimulus type and exposure type across sessions.

Therapist use of pure exposure was primarily used with media versions of vacuum cleaners during sessions 1 and 2 and then used with a toy version and with an actual vacuum cleaner during session 3. Exposure with play and humor was used across all sessions primarily with a toy version and the actual stimulus type, and in shorter durations with media examples in session 1 (see Figure 8). Notably, Danny progressed through his fear hierarchy well, and by session 3, he was able to engage in independent vacuuming (i.e., pure exposure with the actual stimulus) (see Figure 8).

Next, temporal patterns between child and therapist behavior codes were analyzed using lag sequential data analyses. First, temporal patterns between therapist use of invited attention and all child behaviors were analyzed. The results show that the most frequently used therapist technique following child behaviors was invited attention (see Table 7). Specifically, invited attention in session 1 most often followed child tolerating the feared stimulus and child negative behaviors. This demonstrates the therapist working to build and maintain rapport and encourage engagement early in therapy (e.g., redirection and narration) to prepare the child for increasingly challenging levels of the fear hierarchy. In sessions 2 and 3, the therapist most often used invited attention techniques following child tolerant behavior (session 2: 6; session 3: 7) and positive physical child behavior (session 2: 10; session 3: 13) (see Table 7). The pattern of invited attention used in sessions 2 and 3 suggests that as the child progressed through the fear hierarchy and was able to make direct physical contact with the feared stimulus more frequently, the therapist facilitated continued contact and positive engagement through narration, modeling of exaggerated emotion (e.g., “That vacuum is so loud! I'm going to cover my ears!”), and redirection prompts to the exposure. Lag sequential data of child behaviors following invited attention show that this approach yielded some success with Danny. Across sessions, Danny responded to the therapist's use of invited attention with increased frequency of positive physical behaviors (session 1: 5; session 2: 16; session 3: 18) (see Table 6).

## Discussion

In our prior work demonstrating preliminary acceptability and utility of play- and humor-infused exposure therapy for children with Williams syndrome, parent and clinician ratings indicated improvements in fear for some children with Williams syndrome, even after a very brief course of treatment. The current study validated these clinician and parent ratings by demonstrating that this observed improvement was evident based on behavioral coding. Increased tolerance of the feared stimulus across sessions and/or movement from engagement with videos or toys to engagement with real feared objects was observed over the course of this brief intervention for all of the participants. These findings add to our preliminary evidence of the promise of this play- and humor-infused exposure therapy for children with Williams syndrome to address intense emotional dysregulation in response to specific stimuli (i.e., fears and phobias). Additionally, the combination of therapist techniques, including use of invited attention, social play and humor, and relations of these techniques to child behavior, suggests that the therapist's attunement to the child's distress level encourages continued contact with the feared stimulus and results in adjustment of the intensity of the exposure through use of videos, toys, and real-world objects. Findings of the therapist techniques for co-regulation used within this play- and humor-infused approach show that attunement to the child, exaggerated emotion to join the child in their fear, and use of play and humor yield child behaviors suggestive of improved coping through co-regulation, ability to maintain contact with the feared stimulus without becoming overly distressed, ultimately leading to increase in the child's capacity to sustain emotional regulation, improved tolerance, and sometimes positive engagement with their feared stimulus.

The use of humor-infused therapy for children has been suggested to potentially be useful for treatment of anxiety (Consoli et al., 2018) in sustaining emotional regulation in the face of a moderate level of discomfort without overly distressing the child, thus minimizing disengagement from therapy. However, systematic examination of humor-infused therapy is lacking. One critique of this approach is that core to exposure is a need to avoid distractions to facilitate activation and subsequent extinction of the phobic association between the conditioned stimulus and the fear response (Rescorla, 1972). However, we propose that the mechanism of fear reduction here is the establishment and reinforcement of a *new* association of fear inhibition that goes against the typical activation of the amygdala during fear conditioning, rather than simply extinguishing the previously established fear association (Slifer et al., 2002; Craske et al., 2014). Furthermore, behavior therapy has recently been the subject of some critique based on clients' lived experiences due to its perceived aversiveness (Anderson, 2023). Importantly, while the intervention employed in this study is rooted in evidence-based, gold-standard therapies for fears and phobias (APA Division 12 Society of Clinical Psychology, 2022), the therapeutic approach of the current study focused on gradual incorporation of the feared stimulus into play, maintaining the comfort of the child in the context of the exposure to minimize its aversiveness. Essentially, we posit that this intervention fosters formation of an association between the feared stimulus and socially engaging play and humor, which runs counter to the fear response. This model fits well with what is known of the atypical amygdala-prefrontal connectivity thought to be responsible for more pronounced fear associations and subsequent responses (Meyer-Lindenberg et al., 2005).

This play- and humor-infused exposure intervention explicitly uses a therapist–child interactive play-based approach to deliver the therapy to children. The study therapist demonstrated some common patterns related to stimulus type (media, toy, or actual stimulus) and type of exposure (pure exposure or exposure with play and humor). Media examples of the feared stimulus were most often used within pure exposures (i.e., no play or humor). Toy versions of the feared stimulus and the (actual) feared stimulus were typically presented and incorporated within play- and humor-based exposures. This highlights the developmentally attuned nature of the therapeutic approach. By infusing more play and humor into the exposures as the child progresses up the fear hierarchy, the therapist is able to co-regulate with the child and thus the child is able to sustain emotional regulation, minimize distress and anxiety while simultaneously allowing for continued engagement and increasingly more direct contact with the feared stimulus. The feared stimulus is gradually incorporated into the sessions. As designed, this intervention is largely therapist-led, in the presence of the parent. Nevertheless, during this intervention, we observed some parent involvement in the exposures for one participant. It is likely that parental involvement would indeed facilitate generalization of the skills to the home environment. Therefore, future work developing this intervention approach may benefit from more explicit guidance of parents as play- and humor-infused exposure facilitators.

The combination of both behavioral (exposure, systematic desensitization, and counterconditioning) and play-based (age-appropriate social adult–child play development) approaches may be especially well-suited for children with Williams syndrome. The gregarious nature of children with Williams syndrome may mask the underlying emotion dysregulation stemming from anxiety. The focus of this intervention for children with Williams syndrome was to improve emotion regulation relating to specific phobias by building coping skills through social play-based approaches (Farrell et al., 2018) which leverages the social nature and motivation of children with Williams syndrome.

Patterns related to the use of invited attention were also observed. In the context of this study, invited attention was coded when one of the following techniques was observed: priming before an exposure began, narration during or immediately after an exposure, or gesture or verbal prompts during an exposure to direct or redirect the child's attention. Across participants in this sample, the therapist invited attention to the feared stimulus both before and after particular child behaviors were observed. Specifically, the therapist often primed the child before beginning an exposure. The use of narration and exaggerated emotion modeling (e.g., personifying a toy to cry when in the presence of the feared stimulus) was used with all children in this study to encourage continued engagement and promote co-regulation by joining the child in their fear. This approach leverages the empathic and social nature of children with Williams syndrome, by allowing them to experience discomfort and co-regulate with the therapist in a safe and supportive setting. By joining each child in their fear during exposures, the therapist helped them work through these more difficult and uncomfortable emotions (e.g., fear) while gently encouraging their progress through increasingly challenging levels of the fear hierarchy. In some instances, over the course of the intervention, a child even comforted the personified toy (e.g., “it's okay, it's not that loud”). Additionally, when a child's behavior was not clearly positive or negative (i.e., tolerant), the therapist frequently used redirection to prevent escalated anxiety and to promote continued engagement in the exposure activity. The therapist-initiated techniques were often tailored to the individual needs of each child to facilitate progress through the fear hierarchies and ultimately reduce distress in the presence of the phobic stimulus.

Although children with Williams syndrome tend to have auditory-based phobias, the underlying etiology of these sound sensitivities is still undefined. Noise sensitivities may be associated with biological or cognitive behavioral mechanisms. Hyperacusis (i.e., hearing disorder of sensitivity) and phonophobia (i.e., anxiety disorder relating to fear of specific sounds) are two concerns that are commonly expressed by people with Williams syndrome (Silva et al., 2021). Rates as high as 95% (Klein et al., 1990; Nigam and Samuel, 1994) have been reported for people with Williams syndrome and co-occurring hyperacusis, with even more severe symptoms among children (Gothelf et al., 2006). The vulnerability to hyperacusis may be due to the genetic underpinnings of Williams syndrome. Two genes that are deleted on chromosome 7q11.23, one responsible for elastin (ELN) and the other (LIMK-1) dedicated to encoding a serine/threonine kinase, may provide a biologically based explanation for hyperacusis. Deficiencies in elastin may stiffen the stapedius tendon, which is responsible for sound regulation, and may therefore contribute to hyperacusis (Levitin et al., 2005; Prasad et al., 2019). Additionally, the LIMK-1 gene is responsible for the regulation of outer hair cell movement, and dysfunction in this gene has been suggested to increase sound amplification and subsequent startle response to auditory stimuli (Stanyon and Bernard, 1999; Meng et al., 2002; Matsumoto et al., 2011; Tyler et al., 2014). Further research is needed to provide clearer characterization of sound processing abilities and dysfunction among people with Williams syndrome, with particular attention toward the presentation of simultaneous aversion and keen interest in certain sounds (Krilčić and Petranović, 2017). A case study examined the effectiveness of a modified CBT intervention on anxiety and avoidance related to noise sensitivity in an adolescent with autism spectrum disorder and co-occurring intellectual disability (Fodstad et al., 2021). Treatment progress in this study was defined as improved tolerance to auditory input and reduced problem behaviors, with predefined coping skills available for participant use during exposure to such noise. Our current research study aligns with this procedure by allowing participants to engage in simultaneous negative and positive physical behaviors in the presence of the auditory-based feared stimulus (e.g., Beth covering her ears while also approaching a blender turned on), to ultimately increase emotional regulation in the presence of the feared stimulus in naturalistic settings. It is notable that evidence-based treatments for misophonia are largely exposure-based (Bernstein et al., 2013; McGuire et al., 2015; Reid et al., 2016). It may be that developmentally, when the children are young, they may be vulnerable to actual intense discomfort in response to some sounds. Physiologically, this may improve as they physically develop, yet the initial frightening experiences may evolve into an emotionally based phobia, with or without continued sensory sensitivity. Taking sound sensitivities into consideration, it is important to note that the brief play-and humor-infused exposure intervention used in this study demonstrated effectiveness for all four children, regardless of the etiology of such noise sensitivities.

## Limitations and future directions

Limitations include a very small sample size (*n* = 4) and an A-B single-subject research design, which limit the generalizability of findings. This sample is made up of four participants who were White, hence lacked racial diversity. Recruitment strategies to diversify samples in future studies are needed. Furthermore, there were limitations to the behavior coding scheme. Specifically, the behavior coding scheme focused on coding the child's primary fear only (identified during the initial functional assessment interview) and did not capture the intensity of the stimulus sound or the intensity of the child's verbal responses (e.g., volume of protest). Therapist behavior patterns of exposure using sequenced feared stimuli across sessions that included secondary feared stimuli (identified during the functional assessment interview) were not coded (e.g., exposure with the use of a thunder video as a secondary feared stimulus, followed by exposure with the primary feared stimulus of a hand dryer). Furthermore, the behavior coding scheme did not explicitly capture child-initiated coping, which was on occasion observed. For example, Ashton was exhibiting increasing fearful behaviors while the hand dryer was turned on, and he then requested the use of a thunder tube to hold and shift attention toward; after holding and listening to the thunder tube while still in the presence of the hand dryer, his observable fear response reduced. Finally, we acknowledge that behavior coding introduces the possibility of rater bias (i.e., differences between rater identities and participant identities), which we aimed to minimize through independent behavior coding by respective coders.

Further examination of the effectiveness of this approach is needed using a more controlled, multiple baseline research design. Notably, the main aim of this initial round of this research was to gather video illustrations of this play- and humor-infused exposure therapy approach to disseminate to community clinicians; none of the participants completed what would be considered a full round of the intervention. Future research would benefit from collection of more systematic baseline and follow-up data as well as inclusion of community clinicians who can implement the appropriate length of intervention based on the specific needs of the child without the time constraints inherent in the design of the current study. Future studies may also explore associations between the auditory intensity of feared stimuli and the intensity of the child response, or physiological response to the feared stimuli, to better characterize the etiology of sound sensitivities among children with Williams syndrome and provide recommendations on intervention modifications based on co-occurring conditions (e.g., hyperacusis, misophonia, phonophonia, and ASD) and degree of general cognitive and language difficulties. Additionally, the role of parent psychoeducation within this play- and humor-infused exposure therapy may be investigated in future research. Specifically, examination of the effects of parent psychoeducation about mechanisms for fear and anxiety and parental instruction in the use of play- and humor-infused approaches on the sustained impact of the intervention within the naturalistic environment is warranted.

## Conclusion

This study adds to the sparse research on the utility and effectiveness of interventions for children with Williams syndrome and co-occurring fears and phobias. Specifically, the findings of the current study provide support for increased emotion regulation in the form of improved tolerance of the feared stimulus and/or the ability to progress through the fear hierarchy (i.e., media version to toy version to real-world stimulus) following a brief social play- and humor-infused exposure therapy approach with four children with Williams syndrome and co-occurring fears and phobias based on observational behavior coding. For two of the four children, improvements were evident in a move beyond tolerating the feared stimulus toward increased positive behaviors with the feared stimulus across sessions. All four children in this sample progressed through their fear hierarchy and were able to tolerate or even positively engage with the real-world previously feared stimulus by the final therapy session. This line of research provides evidentiary support for developmentally attuned approaches to exposure-based interventions for children with Williams syndrome, adding to the paucity of treatment development literature addressing anxiety and phobias in children with rare neurogenic conditions.

## Data availability statement

The raw data supporting the conclusions of this article will be made available by the authors, without undue reservation.

## Ethics statement

The studies involving human participants were reviewed and approved by University of Wisconsin-Milwaukee Institutional Review Board. Written informed consent to participate in this study was provided by the participants' legal guardian/next of kin. Written informed consent was obtained from the individual(s), and minor(s)' legal guardian/next of kin, for the publication of any potentially identifiable images or data included in this article.

## Author contributions

BY contributed to the development of the coding scheme, served as the primary coder, analysis and interpretation of the data, drafting of the manuscript, and revising the manuscript based on co-author feedback. EM contributed to the refinement of the coding scheme, served as the secondary coder, contributed to data interpretation, and provided conceptual revision feedback on the manuscript. KL originated the humor- and play-infused exposure therapy approach, contributed to the development of the intervention manual, served as the study therapist, and provided conceptual feedback on the manuscript. BK-T collaborated with KL to operationalize the intervention approach into a treatment manual, recruited and enrolled participants, coordinated intervention appointments, assisted with intervention appointments, and supervised the development and implementation of the coding scheme, data interpretation, manuscript-writing, and including providing manuscript revisions. All authors contributed to the article and approved the submitted version.
